# Specificity Evaluation of a Polyprotein‐Based ELISA Designed for the Detection of Paratuberculosis in Multiple Species

**DOI:** 10.1155/vmi/7325758

**Published:** 2026-01-20

**Authors:** R. D. Moyano, M. A. Colombatti Olivieri, M. Basconez González, J. M. Garrido, R. A. Juste, M. N. Alonso

**Affiliations:** ^1^ Agricultural Biotechnology and Molecular Biology Institute (IABIMO), National Institute of Agricultural Technology (INTA), National Scientific and Technical Research Council (CONICET), Hurlingham, Buenos Aires, Argentina, conicet.gov.ar; ^2^ Basque Institute for Agricultural Research and Development (NEIKER), Berreaga 1, Derio, 48160, Bizkaia, Spain

**Keywords:** bovine tuberculosis, cross-reactivity, ELISA, paratuberculosis, polyprotein

## Abstract

Paratuberculosis (PTB) is a disease affecting ruminant animals. The etiological agent, *Mycobacterium avium* subspecies PTB (Map), is a short, Gram‐positive, acid‐fast bacillus. Due to the nature of PTB, diagnosis often occurs at advanced stages of the disease, following the onset of clinical symptoms and prior dissemination of the agent. The specificity of current humoral diagnostic techniques, such as ELISA, is limited, mainly due to the presence of various species of mycobacteria in the environment and other closely related pathogenic mycobacteria that can interfere with the accurate diagnosis of PTB due to the high rate of gene conservation within the genus. In the present study, we evaluated the effectiveness and specificity of an ELISA based on a Map polyprotein for diagnosing bovine PTB. The efficacy of the polyprotein‐based ELISA was assessed using serum samples from healthy, Map‐infected, and *Mycobacterium bovis* (MB)‐infected cattle. Specificity was further evaluated using serum samples from healthy and MB‐infected wild animals. Our findings revealed that in most cases, the degree of cross‐reactivity was negligible or low, particularly in cattle, swine, and goats, while it ranged between 10% and 12.5% in sheep and cervids, respectively. These results suggest that the Map polyprotein used in the ELISA represents a valuable tool for the specific diagnosis of PTB in herds.

## 1. Introduction

Paratuberculosis (PTB) is caused by *Mycobacterium avium* subsp. PTB (Map), a worldwide disease affecting domestic and wild ruminants, leading to economic losses in the dairy and meat industries. Besides ruminants, Map was detected in various wildlife species, including wild boar (*Sus scrofa*) [1]. The clinical signs of PTB are diarrhea, cachexia, submandibular edema, and, in critical situations, it can cause animals’ death is mainly due to extreme weight loss, dehydration, or general deterioration in their health [2]. The primary transmission route is fecal‐oral in approximately 80% of cases; however, other routes of transmission also exist, including the ingestion of contaminated milk or colostrum, as well as artificial or congenital insemination [3].

One of the main challenges for disease control is its chronic nature, as clinical signs usually appear only in advanced stages, hindering early detection and facilitating within‐herd transmission. Another challenge for the control of PTB in herds is the presence of other mycobacteria, such as *Mycobacterium bovis* (MB), which causes bovine tuberculosis, as well as various environmental mycobacteria that can interfere with serological diagnoses of PTB. This interference is mainly attributed to the similarity of the antigenic components shared by related mycobacteria [4–6]. Furthermore, the specificity of serological assays is compromised by cross‐reactions with other non‐mycobacterial species, such as *Nocardia* and *Corynebacterium* species [7–10]. These limitations highlight the reduced specificity and sensitivity in PTB diagnosis, underscoring the need for more precise serological tools. Additionally, when developing detection techniques for the Map, it is crucial to ensure that they do not interfere with bovine tuberculosis diagnostic methods, such as the skin test and its repeated applications in herds, or with animals vaccinated against PTB [11–13]. Therefore, developing a specific diagnostic test for PTB that can be applied in herds to improve disease control is necessary.

In Argentina, the seroprevalence of PTB is estimated to be approximately 30% in dairy herds [14–16]. One of the most common techniques used to detect Map is the enzyme‐linked immunosorbent assay, ELISA. Different ELISA tests have been developed in recent years [17–21] demonstrated that sera from bovine tuberculosis‐infected animals contained high levels of cross‐reactive antibodies and that the commercial IDEXX PTB ELISA was unable to reliably differentiate between PTB and bovine tuberculosis.

Assessing the performance of serological assays across different animal species is crucial to ensure diagnostic reliability under field conditions where PTB often coexists with other mycobacterial infections. In this context, the evaluation of polyprotein‐based ELISA using sera from healthy animals and animals infected with pathogenic mycobacteria provides key insights into potential cross‐reactivity, a major limitation of current diagnostic tools. This approach is particularly relevant in endemic regions, where overlapping infections hinder accurate diagnosis and compromise herd management.

The present study aims to assess potential cross‐reactivity in serum samples from various species of healthy or tuberculosis‐infected animals using a polyprotein‐based ELISA designed to detect Map antigens. This evaluation is intended to determine whether the test can be safely implemented in herds affected by both diseases, a problem frequently observed in numerous herds. Additionally, we evaluated the same serum samples using the commercially available ELISA from the IDEXX company.

## 2. Materials and Methods

### 2.1. Serum Samples

All serum samples used in this study were previously characterized using a commercial, validated ELISA for MB detection, as described in the next section. Based on this initial analysis, the serum samples were classified as positive or negative for tuberculosis and are referred to in this study as +TB or −TB, respectively. The same procedure was then performed using the commercial ELISA for Map antigen detection using the validated PTB IDEXX Map antibody test kit, and is referred to in this study as +PTB or −PTB.

The present study was carried out using 72 (+TB) and 105 (−TB) bovine serum samples, 31 (+TB) and 90 (−TB) swine serum samples, 21 (+TB) and 72 (−TB) goat serum samples, 49 (+TB) and 73 (−TB) ovine serum samples, and 64 (+TB) and 60 (−TB) cervid serum samples. For *M. avium* subsp. PTB 25 (+PTB) and 16 (−PTB) bovine serum samples were used. Aliquots of all sera samples were stored at −20°C until use.

This study was authorized by the Institutional Animal Care and Use Committee (CICUAE) of CICVyA‐INTA, whose regulations comply with the European Union Laws for the protection of experimental animals.

### 2.2. ELISA for the Detection of Antibodies Against Map

#### 2.2.1. Map Polyprotein ELISA

ELISA was used for Map antibodies detection as previously described [20]. Briefly, the Map recombinant polyprotein was expressed in *E. coli* and consists of sequences from four Map antigens from the UniProt database. B‐cell epitopes were predicted using BepiPred‐2.0, and purification was done via nickel affinity chromatography (Qiagen). Polystyrene microtiter ELISA plates (Nunc MaxiSorp, Thermo Fisher Scientific, USA) were coated with 100 μL of carbonate buffer (0.1 M sodium bicarbonate, 0.1 M sodium carbonate, pH 9.6) containing 4 μg of the Map polyprotein and subsequently incubated overnight at 4°C. The wells were then blocked with 0.2% porcine gelatin A (Sigma‐Aldrich, USA) and then washed with PBST. Sera (100 μL/well; 1 : 100 dilution in PBS) were added and incubated for 1 h at 37°C. The wells were washed with PBST before adding peroxidase‐labeled affinity‐purified protein G (Sigma, USA) in a 1 : 12,000 dilution. Finally, the plates were washed, and the reaction was developed using 3,3′,5,5′‐Tetramethylbenzidine (TMB Substrate, Sigma‐Aldrich cat. T0440) and stopped with sulfuric acid 0.2 N (Sulfuric Acid, Sigma‐Aldrich cat. 258105). The optical density (OD) values were measured at 450 nm.

#### 2.2.2. IDEXX PTB Screening ELISA

Additionally, the Map antibody detection in serum samples from both infected and healthy animals was tested using the commercial IDEXX PTB Screening ELISA, based on Institut Pourquier technology, following the supplier’s instructions. In both Map ELISA, the antibody reactivity of each sample was expressed with a corrected OD, OD_450_ (OD at 450 nm obtained in the sample wells minus OD at 450 nm in PBS solution).

### 2.3. ELISA for Detection of Antibodies Against MB

#### 2.3.1. IDEXX MB Ab Test ELISA

The IDEXX MB antibody test was used to detect antibodies against MB in plasma and serum samples from cattle. For the test, a 1 : 200 serum dilution was used, following the manufacturer’s protocol to ensure accuracy and reliability. According to Carneiro et al. [22], this test demonstrates a specificity of 94.7% in detecting MB antibodies in cattle sera. The test has been validated and certified by the World Organization for Animal Health (OIE) and is suitable for the intended purposes, as outlined in the package insert provided with the kit. The registration number indicates the OIE certification 20120107 [23], which demonstrate the test’s international standards and their appropriateness for diagnostic use in the context of bovine tuberculosis.

#### 2.3.2. INgezim Tuberculosis Dual Recognition Immunosorbent Assay (DR‐ELISA)

DR‐ELISA was used to evaluate serum samples from healthy or MB‐infected goats, ovine, swine, and cervids. This kit is an immunoenzymatic assay developed by INGENASA (Madrid, Spain) that detects specific antibodies to MPB83 from MB using a double recognition step (DR‐ELISA). The DR‐ELISA was performed following the supplier’s instructions. The cutoff was calculated according to the manufacturer’s instructions. Samples were considered positive if the OD value at 450 nm was higher than the established positive cutoff threshold [24, 25].

### 2.4. Data Analysis

Two antibodies detection‐based ELISAs were compared for their ability to detect Map‐specific antibodies across several animal species. The sensitivity of the polyprotein ELISA was evaluated using the IDEXX PTB Screening ELISA as the gold standard. Receiver operating characteristic (ROC) analysis was conducted to assess the sensitivity (Se) and specificity (Sp) of the test at different threshold (cutoff) values. The diagnostic value, represented as the sum of sensitivity and specificity ([Se + Sp]/200), along with the specificity discrimination index (SpDI) and sensitivity discrimination index (SeDI), was calculated, along with 95% confidence intervals. The cutoff values that yielded the highest diagnostic value were chosen as the optimal thresholds for each ELISA. These cutoff values were selected from regions where small variations in their numerical values did not significantly alter the sum of sensitivity and specificity.

## 3. Results

### 3.1. Map Polyprotein ELISA Cross‐Reactivity in Bovine Sera

The cross‐reactivity of the polyprotein ELISA was examined using sera from bovines infected with MB. To do this, in the first instance, we determined the cutoff value of the polyprotein‐based ELISA using 34 (+) and 105 (−) sera samples. These data were analyzed using ROC curve analysis to select the optimal cutoff values and to estimate the diagnostic sensitivities and specificities according to each possible cutoff point. Based on this analysis, the estimated specificity was 100%, and the estimated sensitivity was 93.33% of the Map polyprotein ELISA under the tested conditions, and the established cutoff line was 0.1455 (Figure 1(a)).

Figure 1(a) Receiver operating characteristic (ROC) curve analysis of the polyprotein ELISA, constructed using 34 serum samples classified as positive or negative based on the IDEXX Paratuberculosis Screening ELISA results. The table below the ROC curve summarizes the sensitivity, specificity, and likelihood ratios for various cutoff points. The analysis demonstrates the diagnostic performance of the polyprotein ELISA for detecting paratuberculosis (PTB). (b) Dot plot comparing the performance of the polyprotein‐based ELISA developed in this study with the commercial IDEXX PTBC kit for detecting paratuberculosis in bovine samples. The plot shows optical density (OD450 nm) values for TB‐positive animals (*n* = 72) and negative controls (*n* = 105). The dotted line indicates the cutoff value for the polyprotein or IDEXX ELISA.

**Figure:**
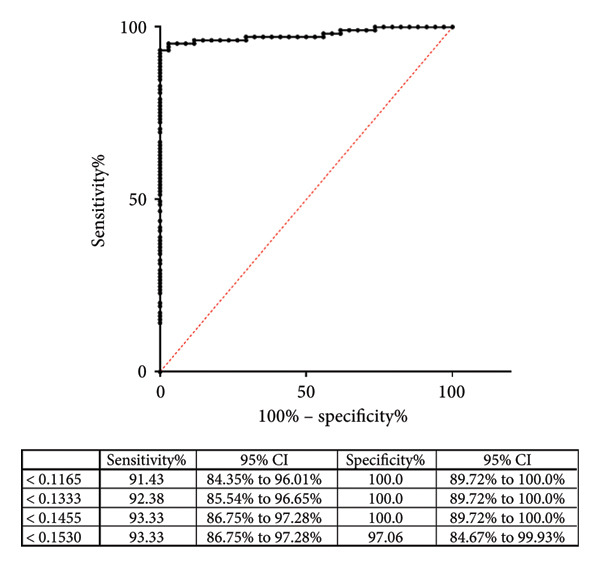
(a)

**Figure:**
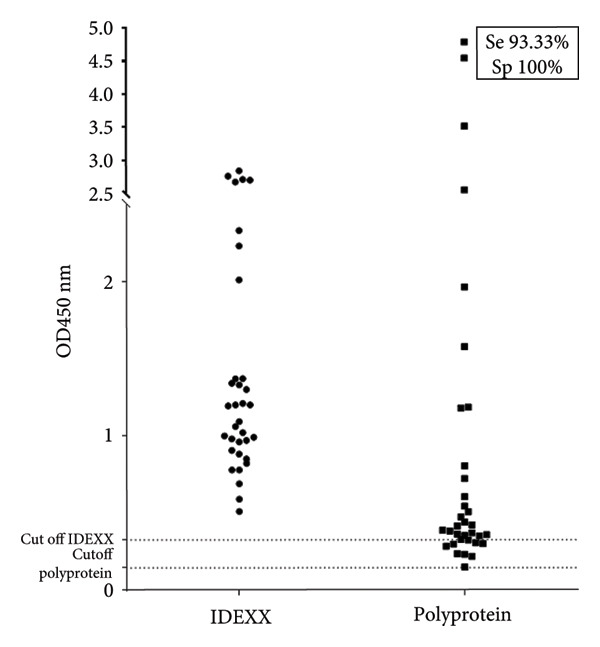
(b)

Once the parameters of the polyprotein‐based ELISA were established, we proceeded to compare the results obtained in the Map polyprotein ELISA with the commercial kit IDEXX PTB Screening ELISAs are considered the gold standard in this work to identify healthy or Map‐infected bovines (+PTB). Thirty‐four serum samples were tested in both polyprotein and commercial ELISA, and we observed that in both tests, the results were identical: all negative or positive serum samples were considered negative or positive in both cases (Figure 1(b)).

The next step was to evaluate bovine serum samples from healthy and bovine TB‐infected animals previously characterized and validated using the commercial ELISA IDEXX MB. In this case, we observed that three serum samples from bovine TB‐infected cattle and seven serum samples from healthy bovines tested positive in the Map polyprotein ELISA, demonstrating a low cross‐reactivity of 6.67% in the TB‐negative group and 5.56% in the TB‐positive group (Figure 2 and Table 1). Reanalysis of the ROC curves, including tuberculosis‐positive animals as the PTB‐negative group, shows a decrease in specificity to 97.73 and a slight increase in sensitivity to 94.35. This shows a robust performance of the technique.

Figure 2Dot plot illustrating the results of the Map polyprotein‐based ELISA performed on bovine serum samples. A total of 177 samples were analyzed, comprising 72 sera from bovines infected with *Mycobacterium bovis* (TB (+)) and 105 from healthy bovines, previously classified using the IDEXX tuberculosis kit. Optical density (OD450 nm) values are shown for each sample, with the dotted line indicating the cutoff value established for the polyprotein ELISA. The test demonstrated a sensitivity of 94.35% and a specificity of 97.73%.

**Figure:**
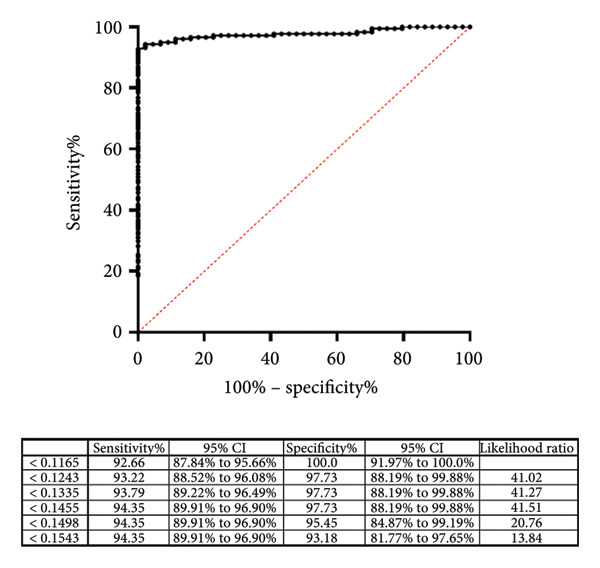
(a)

**Figure:**
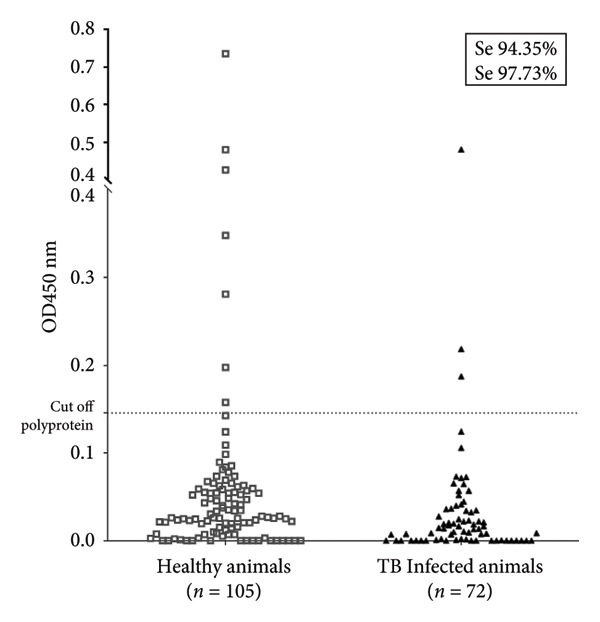
(b)

**Table 1:** Cross‐reactivity of the Map polyprotein ELISA across various species, including bovine, swine, goat, cervid, and ovine.

	Total	Positive	Cross‐reaction percentage
TB (+) bovine	72	4	5.56
TB (−) bovine	105	7	6.67
TB (+) swine	31	0	0.00
TB (−) swine	90	0	0.00
TB (+) goat	21	1	4.76
TB (−) goat	72	0	0.00
TB (+) cervid	64	8	12.50
TB (−) cervid	60	2	3.33
TB (+) ovine	49	5	10.20
TB (−) ovine	73	4	5.48

*Note:* The table summarizes the total number of serum samples tested, the number of positive results obtained using the Map polyprotein ELISA, and the percentage of cross‐reactivity observed within each group of animals classified as either tuberculosis‐positive (TB (+)) or tuberculosis‐negative (TB (−)). The cross‐reactivity percentage represents the proportion of positive results detected in TB‐negative animals for each species.

### 3.2. Map Polyprotein ELISA Cross‐Reactivity in Other Species Sera Infected With MB

We subsequently evaluated the possible cross‐reactivity of the Map polyprotein ELISA in sera from several species of healthy and MB‐infected animals that could act as reservoirs and contribute to the dissemination of the agent. In many cases, TB and PTB could coexist in herds. To do this, we evaluated serum samples from goats, sheep, deer, and swine using the DR‐ELISA commercial kit.

In this case, we observed that the results obtained for healthy and MB‐infected animals varied in each species (Table 1). In the TB‐positive groups, 4/72 bovine samples (5.56%), 1/21 goat samples (4.76%), 8/64 cervid samples (12.5%), and 5/49 ovine samples (10.2%) tested positive. In the TB‐negative groups, cross‐reactivity was minimal, with 7/105 bovine samples (6.67%), no reactivity in swine and goats, and 2/60 cervid samples (3.33%) and 4/73 ovine samples (5.48%) testing positive. In general, the cross‐reactivity observed was low, particularly in most species, with higher rates found in cervids and ovines.

## 4. Discussion and Conclusion

Currently available commercial ELISA tests for *Mycobacterium avium* subsp. PTB (Map) mainly uses purified protoplasmic antigen (PPA), a crude extract containing a mixture of Map proteins. These assays exhibit variable sensitivity and specificity [26, 27]. This variability is largely attributed to antigenic differences between the Map strains used to prepare PPA and the strains prevalent in the regions where the tests are applied [28]. Moreover, cross‐reactivity with other mycobacteria, particularly *M. bovis*, remains a significant diagnostic challenge [29]. Recent research has focused on alternative strategies, including secretory proteins or recombinant protein cocktails, which have been shown to enhance both sensitivity and specificity [30–34].

As reported previously [20], conventional serological assays for Map may show limited specificity when applied to animals co‐exposed to *M. bovis*. Building on these findings, the present study specifically evaluated a Map polyprotein‐based ELISA, focusing on its cross‐reactivity with sera from healthy animals and animals infected with *M. bovis*, which is a relevant scenario in herds where both pathogens coexist. The assay demonstrated very low cross‐reactivity (0%–5.5%) in cattle, pigs, and goats, while slightly higher interference was observed in deer (12.5%) and sheep (10.2%). These results highlight that the polyprotein ELISA maintains high specificity across multiple species, even when co‐infections could complicate serological interpretation. Minor adjustments to cutoff values or sample size may further reduce cross‐reactivity. Although infection status in this study was not confirmed by PCR or culture, the data suggest that observed reactivity is primarily associated with Map‐specific antibodies rather than nonspecific environmental exposure [2, 35].

The implications of these findings are significant for PTB diagnostics. By demonstrating low cross‐reactivity in multiple species, this study supports the use of the Map polyprotein ELISA in herds where Map and *M. bovis* coexist, providing more reliable results than conventional assays. Furthermore, its broad applicability across livestock and wildlife enhances its potential for population‐level surveillance and herd management programs, contributing to improved disease control and informing vaccination strategies [36].

The Map polyprotein‐based ELISA represents a valuable advancement in PTB diagnostics, combining low cross‐reactivity with applicability across multiple species. This study demonstrates its potential utility for field implementation in mixed‐pathogen settings, offering improved specificity and practical relevance for herd management and public health initiatives. Further validation with larger and more diverse sample sets will refine performance and establish optimal diagnostic thresholds.

## Conflicts of Interest

The authors declare no conflicts of interest.

## Funding

This study was supported by the Consejo Nacional de Investigaciones Científicas y Técnicas, 10.13039/501100002923, PIP 11220200102994CO.

## Data Availability

The data that support the findings of this study are available on request from the corresponding author. The data are not publicly available due to privacy or ethical restrictions.
